# A Single Nucleotide Polymorphism in the Phospholipase D1 Gene is Associated with Risk of Non-Small Cell Lung Cancer

**Published:** 2012-06

**Authors:** Myung-Ju Ahn, Shin-Young Park, Won Kyu Kim, Ju Hwan Cho, Brian Junho Chang, Dong Jo Kim, Jin Seok Ahn, Keunchil Park, Joong-Soo Han

**Affiliations:** 1 *Biomedical Research Institute and Department of Biochemistry and Molecular Biology, College of Medicine, Korea;*; 2 *Department of Anatomy and Cell Biology, College of Medicine, Hanyang University, Seoul, 133-791, Korea;*; 3 *Division of Hematology-Oncology, Department of Internal Medicine, Department of Medicine, Samsung Medical Center, Sungkyunkwan University, School of Medicine, Seoul 135-710, Korea;*; 4 *John Muir College, University of California, San Diego, 9500 Gilman Dr., La Jolla, CA 92092, USA;*; 5 *Department of Anatomy and Neurobiology, Washington University School of Medicine, 660 S. Euclid Avenue, St. Louis, MO 63110, USA*

**Keywords:** biomarker, DGGE, lung cancer, NSCLC, phospholipase D, single nucleotide polymorphism

## Abstract

Phospholipase D (PLD) has an important role in various biological functions including vesicular transport, endocytosis, exocytosis, cell migration, and mitosis. These cellular biological processes are deregulated in the development of various human tumors. In order to explore the relationship between the PLD1 gene and risk of non-small cell lung cancer (NSCLC), single nucleotide polymorphisms (SNP) in the PLD1 exon region were surveyed in 211 NSCLC patients and 205 normal controls. In this study, we identified six SNPs at exon 23 in the PLD1 gene. Among the six SNPs, the most notable was a heterozygous A to C transition at nucleotide 2698 (A2698C, *p*<0.001). In addition, the genotype frequencies of A2744C (AC+CC) and A2756C (AC+CC) were associated with gender (female, A2744C and A2756C: *p*=0.071) in NSCLC patients. Interestingly, although the SNP A2698C did not cause change in amino acid, correlation between odd ratio of NSCLC patients and the SNP A2698C was observed to be statistically significant.

## INTRODUCTION

Phospholipase D (PLD) is a ubiquitous enzyme that catalyzes the hydrolysis of phosphatidylcholine (PC) to phosphatidic acid (PA) and choline (1). PLD has two isoforms, PLD1 and PLD2, which differ in their mechanisms of activation and subcellular localization (2). In numerous human cancers, both PLD expression and activity are aberrantly increased (3-5). Recently, several studies have shown that expression levels of PLD1 mRNA and protein as well as PLD activity were markedly increased in human breast cancer tissue (6) and human renal cancer (7). We have previously shown that PLD and molecules involved in PLD signaling can be valuable targets for therapeutic intervention in cancers (1). These observations strongly imply that PLD is implicated in tumorigenesis, but the molecular mechanism remains unknown.

Lung cancer is the leading cause of cancer mortality in Korea and worldwide (8-11). Non-small cell lung cancer (NSCLC) accounts for ~85% of all cases of lung cancer (12). Polymorphisms in genes coding for enzymes involved in the metabolic activation and detoxification of tobacco carcinogens in the repair of DNA damage have been associated with an increased risk of lung cancer in case-control studies (13-15). Molecular epidemiologic studies have reported relationships between lung cancer and polymorphisms in genetic susceptibility genes, including metabolic enzymes (cytochrome P450s, glutathione S-transferases) and DNA repair enzymes (hOGG1, XRCC1), with the goal of elucidating their relationships with lung cancer susceptibility (13, 15, 16). Furthermore, a possible association between cancer susceptibility and variation in genes involved in chromatin structure and histone methylation has been investigated (17-19), suggesting that genetic susceptibility plays an important role in lung carcinogenesis.

This study was undertaken to examine the possible relationships between six novel SNPs found in the PLD1 exon 23 region and the risk of NSCLC in Korean NSCLC patients. We observed one and two other SNPs are associated with susceptibility and gender, respectively. Particularly, SNP A2698C leads us to conclude that SNPs on PLD1 may contribute to genetic susceptibility to NSCLC.

## METHODS

### Patients

This study evaluated 211 patients diagnosed with NSCLC who underwent surgical resection at Samsung Medical Center between 2005 and 2008. For comparison, a total of 205 control subjects were individually matched with lung cancer patients for age (± 10 years) and gender (Table 1). The control subjects had no prior histories of cancer and were recruited from a pool of visitors to our institution who were participating in a cancer-screening program. Information on demographic characteristics, including gender, age, and smoking habits, was obtained from self-administered questionnaires (for controls) or personal interviews (for cases) administered by a trained personnel after written informed consent was obtained. This study was performed after approval by the institutional review board at Samsung Medical Center (Seoul, Korea).

**Table 1:** Characteristics of non-small cell lung cancer patients and controls

Variable	Controls	Non-small cell lung cancer cases (n=211)

Age (years)	48.3 (± 10)	59.8 (± 10)
Sex		
Male	124 (60.5%)	139 (66.8%)
Female	81 (39.5%)	72 (33.2%)
Smoking status		
Current	87 (42.5%)	102 (48.3%)
Never	118 (57.5%)	109 (51.7%)

### DNA extraction and PCR amplification

Whole blood samples (1 ml) from the 416 subjects were collected in EDTA tubes. Genomic DNA was extracted using a Puregene DNA Purification kit (Gentra, MN, USA). The 26 exons of the PLD1 gene were amplified by PCR using primers synthesized according to sequences derived from GenBank (NM_001130081) (Table 2). For a standard 30-cycle amplification was performed using the following conditions: 94°C for 1 min, 55°C for 1 min, 72°C for 1 min (for exons 1-14, 18, and 20) or 94°C for 1 min, 60°C for 1 min, 72°C for 1 min (for exons 15-17, 19, and 21-26).

**Table 2:** List of Primers for Detection of SNPs in PLD1 gene

Exon No.	Direction	Sequences	Exon No.	Direction	Sequences

1	Forward	5’-[40GC]GCCCTTTGCTTTTACTCTGTC-3’	14	Forward	5’-[40GC]CATGTCTTATGCAGTGTCTTTT-3’
	Reverse	5’-CGCTCAGATCATCCGTCTTTAC-3’		Reverse	5’-GATAAATTCTAGTCAAGGCCA-3’
2	Forward	5’-AGTGTATATCCCTTTCTCTGC-3’	15	Forward	5’-GATCAGCTTTGCTTTCCAGTTT-3’
	Reverse	5’-GAGTCCATAAACGCTCTGAC[40GC]-3’		Reverse	5’-TAAGGGAGTTCTGCCACTTCA[40GC]-3’
3	Forward	5’-[40GC]ATGTATCACTGTAGGTACCAAG-3’	16	Forward	5’-ACTCACCTGAACCACAGTGT-3’
	Reverse	5’-AAATGGTTACCTTCTAGTGGG-3’		Reverse	5’-AATATAACCAGCACCCCACCA[40GC]-3’
4	Forward	5’-[40GC]GGTGTTTGCATTCTGTGTGT-3’	17	Forward	5’-[40GC]AGACTTTGCCCCAACACTGAA-3’
	Reverse	5’-CTTACTCTTCTACCAAGGAATA-3’		Reverse	5’-GATAAATCATGATAGCAACATCC-3’
5	Forward	5’-[40GC]GATCTCATCATTGTCACTACTG-3’	18	Forward	5’-CACAAAGTAGGGAGAATGAATC-3’
	Reverse	5’-TGACTAGTACTTACTGTGGCAT-3’		Reverse	5’-AAGGGAAGGCAGTTTCTCACA[40GC]-3’
6	Forward	5’-TGAATTGTTTTGCTTGCAAAAG-3’	19	Forward	5’-[40GC]CTGATGTCCTCTCCATTGCTAA-3’
	Reverse	5’-ATGGCATGCTGCTACGTTA[40GC]-3		Reverse	5’-AAGGGAAGTCTAGTAGGTGG-3’
7	Forward	5’-[40GC]TGTTGGGAGGCTGTACGAG-3’	20	Forward	5’-CAGTATTGTTCTTACGTATATTGC-3’
	Reverse	5’-GTAAAACTAGCCCAAATACC-3’		Reverse	5’-AATACAAGAACATCTGCAGCGA[40GC]-3’
8	Forward	5’-[40GC]CTTACTACCTTCTTACAGATGG-3’	21	Forward	5’-[40GC]TGAACTGCTTGGCTGTCATCTA-3’
	Reverse	5’-CCTTGAAAGATTATCAATTCGG-3’		Reverse	5’-ATGATGCATGACCGAAAGCTCA-3’
9	Forward	5’-[40GC]TGAGATAGAACAGAGTGACC-3’	22	Forward	5’-AGGATTAAACCTACAGATACTGC-3’
	Reverse	5’-ATTAGATGCTATGACTGCCCTTG-3’		Reverse	5’-ATGATTACTGATACCTCACCTTC[40GC]-3’
10	Forward	5’-[40GC]TGGAGATCTAGGCAGTGG-3’	23	Forward	5’-[40GC]TGTACGTTTATTGAGCTTGGTCA-3’
	Reverse	5’-GTGTAATTCTTGAACAGCACTA-3’		Reverse	5’-TAAGTCAACTGGCAAGGAATACA-3’
11	Forward	5’-CCTTTGCTTCCTATGACACA-3’	24	Forward	5’-[40GC]GTTGTTCAGCCTCACTGTTTCT-3’
	Reverse	5’-GTGTAATTCTTGAACAGCACTA[40GC]-3’		Reverse	5’-TGGAAATGCATCAGAGAGACAC-3’
12	Forward	5’-[40GC]CGGTTTTCTCCTGTGACAG-3’	25	Forward	5’-CAGTTACTCAATGTGGAGGTCA-3’
	Reverse	5’-ATCAAGATGAACCTGAATACC-3’		Reverse	5’-GAGGAGGGGAATACGTGAACT[40GC]-3’
13	Forward	5’-[40GC]TTTCAGGGAGAACACAGACC-3’	26	Forward	5’-TAAAGGCCATGTGCTCGCTT-3’
	Reverse	5’-GGTTAAATATACTTACTGGGAGG-3’		Reverse	5’-TGGAAGTCTTTGAGCTGCCAA[40GC]-3’

### Denaturing gradient gel electrophoresis (DGGE)

After performing of DGGE to screen whether the sample has SNP, PCR products were loaded on a 20 × 27 cm, 0.75 mm-thick polyacrylamide gel (acrylamide:bisacrylamide, 37.5:1) containing a linear denaturing gradient (100% UF = 7 M urea/40% deionized formamide). The percentage of polyacrylamide varied between experiments. A 9% polyacrylamide stacking gel was poured to create solid slots for efficient loading of the PCR products, preventing difficulties caused by high urea concentration. Electrophoresis was performed in 1 × TAE buffer (40 mM Tris-acetate, 20 mM sodium acetate, 1 mM EDTA, pH8.0) at 59°C. For all experiments performed in this study, fresh buffer was used and only a single experimental condition was changed per test. Time-travel parallel DGGE was performed according to an established protocol. Gels were stained with ethidium bromide and photographs were taken under a UV transilluminator.

### Detection and genotyping of PLD1 polymorphisms

To identify novel polymorphisms in PLD1, DGGE was performed for screening of two or multi bands on 26 exons of PLD1 prior to sequencing. We found several SNPs on exons of PLD1 through direct sequencing using PCR product. Among these SNPs we focused on exon 23 region of PLD1 within a 210 kb segment of genomic DNA. 147 out of 211 NSCLC patients and 77 out of 205 controls were examined by PCR and then analyzed by direct sequencing. If there was no detection of multi bands on DGGE, we considered it as a wild type PLD1. Variants were identified by comparison with traces of the PLD1 sequence relative to the reference GenBank sequence (NM_001130081) and confirmed by re-amplification and re-sequencing. The genotypes of PLD1 SNPs were identified by direct sequencing; PCR primers for PLD1 SNPs at exon23 were as follows: forward: 5’-TGTACGTTTATTGAGCTTGGTCA-3’; reverse: 5’-TAAGTCAACTGGCAAGGAATACA-3’.

### Immunohistochemistry staining

The tissues were fixed overnight in 4% paraformaldehyde at 4°C, deparaffinized, rehydrated and immersed in normal goat blocking serum. The rabbit anti-human PLD1 antibody was used as primary antibody. The secondary antibody was biotinylated goat anti-rabbit antibody and the detection kit was DAB (3, 3′-diaminobenzidine). The assay was repeated five times on each kind of tissues. The immunostaining slides were examined under light microscopy and the digital images were captured with a Leica DM5000B digital camera.

### Statistical analysis

The relationships between clinical factors and PLD1 SNPs were analyzed using the χ^2^-test. The association between NSCLC patients and each individual SNP was estimated using unconditional logistic regression after adjustment for clinical characteristics. The probability of survival was estimated using the Kaplan-Meier method. Differences in survival were evaluated using the log-rank test. All *p*-values were two-sided, and *p*-values less than 0.05 were considered to be statistically significant. Statistical analyses were performed using SPSS software version 13.0 (SPSS Institute Inc., Cary, NC).

## RESULTS

### Identification of PLD1 polymorphisms

**Figure 1:**
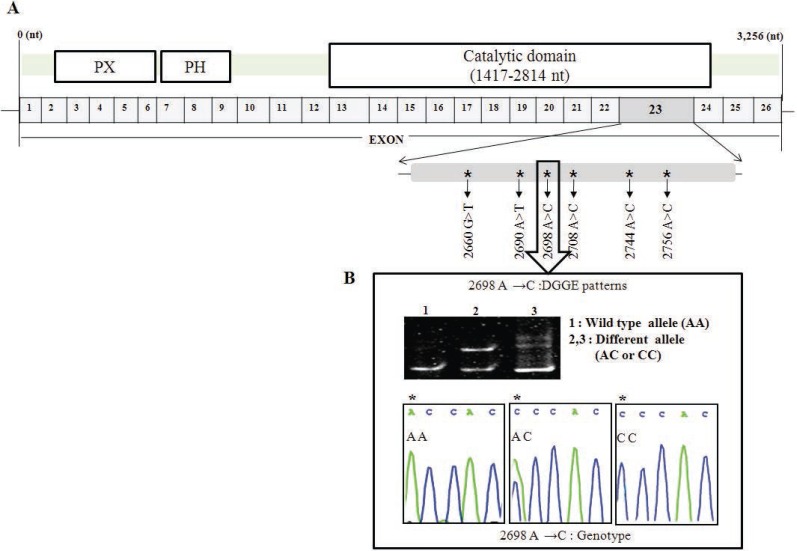
Locations of the six observed SNPs in PLD1 cDNA. (A) Distribution of single nucleotide polymorphism in the PLD1 gene. Substituted nucleotides are indicated by an arrow; (B) Top: DGGE pattern of A2698C showing a different allele (lanes 2 and 3). Bottom: DNA sequencing electropherograms of wild type (AA) and different alleles (AC and CC) of A2698C identified in PLD1 SNPs in this study. Polymorphic sites are indicated by asterisks (*).

We included 211 NSCLC patients and 205 controls in this analysis (Table 1). To identify novel polymorphisms in PLD1, DGGE was performed for screening two or multi bands for every 26 exons of PLD1. For tissues that showed multi bands, direct sequencings were performed. After screening, we found several SNPs on PLD1. Among those SNPs we focused on exon 23 of PLD1, since it has 6 SNPs on a single exon and the frequency of these SNPs in NSCLC patient was high enough to deep search. If there was no detection of multi bands on DGGE, we considered it normal as a wild type of PLD1. We identified six genetic variants by direct DNA sequencing analysis and DGGE within the full 3.2 kb genome, including the exon 23 region (2612 bp ~ 2863 bp) of PLD1: 2660 G→T, 2690 A→T, 2698 A→C, 2708 A→C, 2744 A→C and 2756 A→C. These are novel SNPs within the 26 exons of the PLD1 gene (Figure 1). There were frequency differences in the distribution of variant alleles of the six SNPs between NSCLC patients and controls. Of the novel SNPs, the most notable SNP was a heterozygous A to C transition at nucleotide 2698 (exon 23, allele A/C: A2698C, *p*<0.001, OR=4.619) (Table 3).

**Table 3:** Allelic frequencies of PLD1 SNPs in non-small cell lung cancer patients and controls

Polymorphism	Non-small cell lung cancer (n)	Controls (n)	*P value (n)* (*p*<)	Adjusted OR

2660 G>T	0.94% (2/211)	0% (0/205)	0.246	0.615
2690 A>T	1.44% (3/211)	0% (0/205)	0.195	0.784
2698 A>C	65.9% (139/211)	37.6% (77/205)	0.001	4.619
2708 A>C	0.47% (1/211)	0%	0.437	0.511
2744 A>C	0.47% (1/211)	0%	0.437	0.511
2756 A>C	0.47% (1/211)	0%	0.437	0.511

*p*-values and odd ratio (OR) for allelic frequencies were obtained from Pearson’s χ^2^-test.

### Genotyping SNPs

The genotypic distributions of the six PLD1 SNPs in NSCLC and controls are summarized in Table 4. Even though the number of sample is not enough to define whether they are significantly associated with NSCLC, there were differences in genotype distributions G2660T (*p*=0.013), A2698C (*p*<0.001), A2744C (*P*=0.005), and A2756C (*p*=0.005) between NSCLC cases and controls. However, there was no significant difference in the genotype frequencies of A2690T (*p*=0.092) and A2708C (*p*=0.612) between NSCLC cases and controls.

**Table 4:** Genotype distribution of the PLD1 polymorphisms in non-small cell lung cancer and controls

SNP	Group	Genotypes of PLD1 polymorphisms			
		W/S (%)^a^	W/V (%)^a^	V/V (%)^a^	*P*-value (*p*<)

2660 G→T	Lung cancer	192 (91.0)	17 (8.1)	2 (0.9)	0.013
	control	200 (97.6)	5 (2.4)	0 (0.0)	
2690 A→T	Lung cancer	190 (90.0)	19 (9.0)	2 (0.9)	0.092
	control	195 (95.1)	10 (4.9)	0 (0.0)	
2698 A→C	Lung cancer	50 (23.7)	120 (56.9)	41 (19.4)	0.001
	control	113 (55.1)	69 (33.7)	23 (11.2)	
2708 A→C	Lung cancer	190 (90.0)	20 (9.5)	1 (0.5)	0.612
	control	186 (90.7)	19 (9.3)	0 (0.0)	
2744 A→C	Lung cancer	184 (87.2)	27 (12.8)	0 (0.0)	0.005
	control	195 (95.1)	10 (4.9)	0 (0.0)	
2756 A→C	Lung cancer	184 (87.2)	27 (12.8)	0 (0.0)	0.005
	control	195 (95.1)	10 (4.9)	0 (0.0)	

*p*-values for genotypes was obtained from Pearson’s χ^2^-test.

a W means wild-type allele and V means variant allele of each SNP.

### Association analysis between PLD1 SNPs and clinical characteristics in NSCLC patients

We investigated whether PLD1 SNPs are related to clinical characteristics using logistic regression analysis. Of the six PLD1 SNPs we studied, A2698C (AC+CC) was the only genotype that was highly associated with survival in NSCLC patients (carrying one variant allele vs. none, *p*=0.012) (Table 5). This result indicates that the A2698C PLD1 SNP may be related to risk of NSCLC. The genotypes A2744C and A2756C (AC+CC) were associated with gender in NSCLC patients, although the *p* value did not reach statistical significance (*p*=0.071).

**Table 5:** Association analysis among PLD1 SNPs, smoking status, age and gender in non-small cell lune cancer patients

SNP	Geno-type	Gender (%)			Survival (%)			Smoking (%)			Age-years (%)		
		Male	Female	*P*	Death	N/D^a^	*P*	Never	Ever	*P*	Age ≥60	Age <60	*P*

G2660T	GG	125 (89.9)	67 (93.0)	0.578	88 (82.3)	104 (91.2)	0.967	94 (92.2)	98 (89.9)	0.648	106 (91.4)	86 (90.5)	0.642
	GT+TT	14 (10.1)	5 (7.0)		9 (17.7)	10 (8.8)		8 (7.8)	11 (10.1)		10 (8.6)	9 (9.5)	
A2690T	AA	123 (88.5)	67 (93.0)	0.150	88 (82.3)	102 (89.5)	0.991	92 (90.2)	98 (89.9)	0.932	107 (92.2)	83 (76.3)	0.131
	AT+TT	16 (11.5)	5 (7.0)		9 (17.7)	12 (10.5)		10 (9.8)	11 (10.1)		9 (7.8)	12 (23.7)	
A2698C	AA	34 (24.4)	16 (22.2)	0.536	14 (14.4)	36 (31.6)	0.012	21 (20.6)	29 (26.6)	0.481	29 (25.0)	21 (22.1)	0.369
	AC+CC	105 (75.6)	56 (77.8)		83 (85.6)	78 (68.4)		81 (79.4)	80 (73.4)		87 (75.0)	74 (77.9)	
A2708C	AA	127 (91.4)	63 (87.5)	0.369	88 (90.7)	102 (89.5)	0.626	100 (91.7)	90 (88.2)	0.372	103 (88.8)	87 (91.6)	0.331
	AC+CC	12 (8.6)	9 (12.5)		9 (9.3)	12 (10.5)		9 (8.3)	12 (11.8)		13 (11.2)	8 (8.4)	
A2744C	AA	117 (84.2)	67 (93.0)	0.071	83 (73.2)	101 (88.6)	0.661	94 (86.2)	90 (88.2)	0.662	105 (90.5)	79 (83.2)	0.236
	AC+CC	22 (15.8)	5 (7.0)		14 (26.8)	13 (11.4)		15 (13.8)	12 (11.8)		11 (9.5)	16 (16.8)	
A2756C	AA	117 (84.2)	67 (93.0)	0.071	84 (86.6)	100 (87.7)	0.651	96 (88.1)	88 (86.3)	0.628	99 (85.3)	85 (89.5)	0.528
	AC+CC	22 (15.8)	5 (7.0)		13 (13.4)	14 (12.3)		13 (11.9)	14 (13.7)		17 (14.7)	10 (11.5)	

*p*-values for gender, survival, smoking and age were obtained from logistic regression analysis.

a Non Death.

### Expression of PLD1 in NSCLC

**Figure 2:**
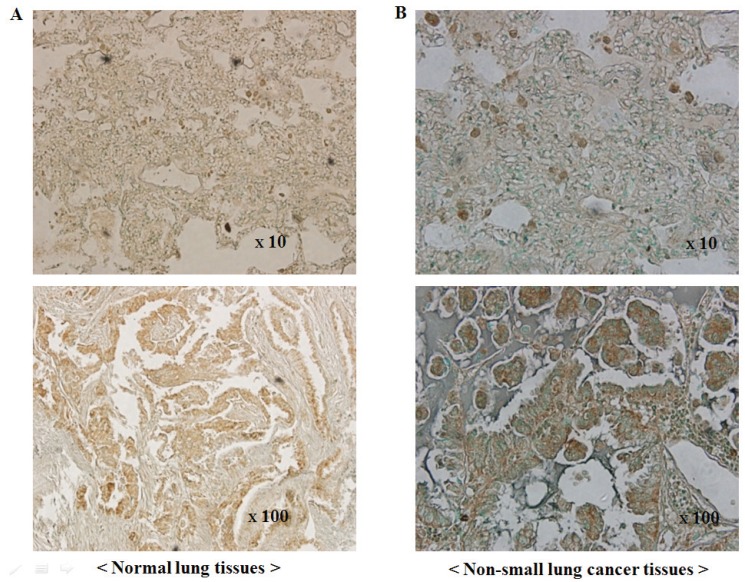
PLD1 immunohistochemistry in human non-small cell lung cancers and normal lung tissues. (A) Immunoreactivity for PLD1 in normal alveolar lung tissues. The acinar epithelia and intra-alveolar connective tissue exhibit a negative reaction and positive staining, respectively; (B) Immunoreactivity for PLD1 in non-small cell lung cancer tissues (NSCLC). NSCLC tissues exhibit a strong positive reaction (top: ×10, bottom: ×100).

The expression of PLD1 protein was examined by immunohistochemical staining using surgical specimens from an independent set of 215 NSCLC patients (Figure 2). Expression was detected in 179 (83.3%) of 215 NSCLC tissues, while PLD1 was not readily detected in normal lung tissues. However, there was not a significant difference in overall survival between the PLD1-positive and the PLD1-negative group in NSCLC patient (data not shown).

## DISCUSSION

Individual susceptibility to lung cancer has been shown to vary with the presence of single nucleotide polymorphisms (SNPs) in a number of critical genes (20-22). It is quite possible that DNA sequence variations in PLD1, a gene that has recently been studied for its association with the development of many cancers, may lead to alteration in the activity of PLD, which can cause individual differences in lung cancer susceptibility. Indeed, PLD is a critical regulator of cell proliferation, survival, and abnormalities in many cancer cells, such as PC 12 cells, v-*src*-transformed rat fibroblasts, and MDA-MB-231 breast cancer cells (6, 23-25). Recently, it has been reported that PLD and molecules involved in PLD signaling may be valuable targets in therapeutic interventions for cancers, given that a substantial portion of tumor cells apparently has elevated PLD activity (1). Furthermore, activation of PLD1 by bradykinin and sphingosine 1 is involved in the protein kinase C signaling pathway in A549 human lung adenocarcinoma cells (26, 27), suggesting a possible association between PLD1 and NSCLC. Recently, it has been reported that PLD polymorphisms are closely associated with cancer. For examples, the C1814T (Thr577Ile) polymorphism in the human PLD2 gene is associated with the prevalence of colorectal cancer (28), and a naturally-occurring variant of human PLD2 in which Gly901 in the COOH-terminal region is replaced by the charged amino acid Asp is catalytically inactive (29).

Interestingly, the six PLD1 SNPs in the present study were located in the catalytic domain of the PLD1 gene (Figure 1). We analyzed whether six PLD1 SNPs (G2660T, A2690C, A2698C, A2708C, A2744C, and A2756C) function as a biomarker which contributes to prediction or risk of NSCLC based on the results from direct sequencing and DGGE of the whole genomic region in 211 Korean NSCLC patients and 205 normal controls. Even though tobacco smoking has a well-established critical role in the development of lung cancer, association study of PLD1 SNP genotypes using smoking status and age in NSCLC patients did not show any significant correlation with smoking status (Table 5). However, the variant alleles A2744C (AC+CC) and A2756C (AC+CC) have correlation with gender, particularly female NSCLC group (*p*=0.072), although this relationship was not statistically significant. Among the six SNPs, the variant (AC+CC) A2698C SNP was the only genotype associated with an increased risk of developing NSCLC. Unfortunately, A2698C is a synonymous mutation (GTA^val^ → GTC^val^); however, it is reported that altering nucleotide between the same amino acids does affect tRNA affinity during translation. According to Elf and Nilsson *et al*’s reported that GTAval increased tRNA affinity to mRNA more than GTC^val^ (30), PLD expression level should be decreased in NSCLC patients. However, as shown in Figure 2 and Table 3, PLD expression level and PLD SNP occurrence in NSCLC patients are higher compared to normal. Indeed, these findings suggest that there is a possible mechanism for regulation of PLD expression or maintenance. To the best of our knowledge, this is the first study to demonstrate a statistically significant association between PLD1 SNPs and NSCLC risk. Furthermore, it is the first to show that one of the PLD SNPs, A2698C, causes an increase in NSCLC susceptibility. These results suggest that the presence of A2698C SNP in PLD1 may be involved in the development of NSCLC and could be one of important markers of genetic susceptibility to lung cancer. Precise mechanism by which susceptibility to NSCLC increases remains uncovered, yet.
